# Identification of Aroma Compounds of *Lamiaceae* Species in Turkey Using the Purge and Trap Technique

**DOI:** 10.3390/foods6020010

**Published:** 2017-02-08

**Authors:** Ahmet Salih Sonmezdag, Hasim Kelebek, Serkan Selli

**Affiliations:** 1 Department of Gastronomy and Culinary Arts, Faculty of Fine Arts, Gaziantep University, Gaziantep 27100, Turkey; sonmezdag@gantep.edu.tr; 2 Department of Food Engineering, Faculty of Engineering and Natural Sciences, Adana Science and Technology University, Adana 01100, Turkey; hkelebek@adanabtu.edu.tr; 3 Department of Food Engineering, Faculty of Agriculture, Cukurova University, Adana 01330, Turkey

**Keywords:** aroma, purge and trap, *Salvia officinalis*, *Lavandula angustifolia*, *Mentha asiatica*

## Abstract

The present research was planned to characterize the aroma composition of important members of the *Lamiaceae* family such as *Salvia officinalis*, *Lavandula angustifolia* and *Mentha asiatica*. Aroma components of the *S. officinalis*, *L. angustifolia* and *M. asiatica* were extracted with the purge and trap technique with dichloromethane and analyzed with the gas chromatography–mass spectrometry (GC–MS) technique. A total of 23, 33 and 33 aroma compounds were detected in *Salvia officinalis*, *Lavandula angustifolia* and *Mentha asiatica*, respectively including, acids, alcohols, aldehydes, esters, hydrocarbons and terpenes. Terpene compounds were both qualitatively and quantitatively the major chemical group among the identified aroma compounds, followed by esters. The main terpene compounds were 1,8-cineole, sabinene and linalool in *Salvia officinalis*, *Lavandula angustifolia* and *Mentha asiatica*, respectively. Among esters, linalyl acetate was the only and most important ester compound which was detected in all samples.

## 1. Introduction

Throughout human history, medicinal and aromatic plants have been used for flavor enrichment in culinary and medicinal purpose in folk medicine. Today, the usage of these plants in daily diet has increased significantly all over the world. Within this trend, the family of *Lamiaceae* has been of great importance due to the unique aroma and nutritional value [1]. *Lamiaceae* is one of the widespread and the most exclusive medicinal and aromatic family of flowering plants, containing about 220 generas and around 4000 species all over the world. Generally, this family is cultivated in the dry, mild, and cold districts of Asia, Europe and North Africa [2]. Archeological excavations showed that the usage of this family member is based on prehistoric times and harvested not only wild but also in local balances [3]. This great diversity includes the following species: lavenders, sage, and mints.

One of the most important members of the *Lamiacae* family is Lavenders (*Lavandula* spp.). This genus is native and widely distributed in the Mediterranean region. *Lavandula* contains sesional, medicanal shrubs and small herbs, which have aromatic parts [4]. *Lavandula angustifolia* is an endemic, widely distributed and taxon of the Mediterranean part of Turkey. It has a great market value owing to the strong and characteristic aroma. Thus, dry herb or essential oil of the plant is very demanded in flavoring, pharmaceutical, and food industries [5]. The *Salvia* L. (sage) is one of the main genera of the *Lamiaceae* family and contains almost 900 species all over the world, of which 94 taxa, belonging to 89 species, are grown in Turkey. *Salvia officinalis* L. is also endemic to the Mediterranean districts as a seasonal medicinal and aromatic herb. The plant has been widely used traditionally in food preparation, flavoring agents in perfumery, and cosmetics. Additionally, it has been used for medicinal purposes for a long list of diseases [6]. The *Mentha spp.* is another famous essential oil herb of medicinal and aromatic plants and is an important commodity owing to the huge requisitions for its volatiles oil in foodstuff, medicinal and hygiene manufactures. Globally, the genus Mints consist of 62 taxa and 18 species. Furthermore, mints have been consumed in folk medicine for the treatment of many complaints owing to its anti-inflammatory, analgesic, and sedative effects [7,8].

The volatile composition of *Lamiaceae* is affected by several different and very heterogeneous chemicals (e.g., alcohols, aldehydes, esters, ketones, acids, terpenes, etc.). Some of the short chain terpenes that constitute the main fraction of the *Lamiaceae* family, especially C10 mono- and C15 sesquiterpenes, overwhelmingly affect the flavor and taste of this family [9]. This group includes some pleasant smelling volatiles and terpenes-rich herbs which are very important in culinary and perfumery industry. In addition, many of its members have anti-bacterial effects and these specialties are mainly owing to the C10 mono- and C15 sesquiterpenes in the herb [10]. To the best of our knowledge, there is some research regarding the essential oil of these three members of *Lamiaceae* but no combined clarification of information from GC and gas chromatography–mass spectrometry (GC–MS) analysis, has been conducted on the aroma composition of Turkish origin.

Therefore, the aim of the present research was to identify and quantify the volatile composition of three members, *Salvia officinalis*, *Lavandula angustifolia* and *Mentha asiatica*, of the *Lamiaceae* family, all of which are cultivated in Turkey. In the present study, the aroma extraction method selected was the purge and trap technique with dichloromethane solvent. This technique is a very sensitive extraction method for many aroma compounds, especially with low boiling points. Additionally, by using this technique, it is possible to extract volatile compounds without artifacts formation with high reliability gas chromatography (GC) together with mass spectrometry (MS) and a flame ionization detector (FID) for quantification and identification of volatile compounds.

## 2. Materials and Methods

### 2.1. Samples and Chemicals

Commercial samples (1 kg) of dried young leaves of *Salvia officinalis*, *Lavandula angustifolia* and *Mentha asiatica* (origin: Turkey) were obtained from a local herbalist supplier, in Gaziantep, Turkey in July 2016. The herbs were identified by the Faculty of Agriculture, University of Cukurova. The moisture content of the herbs was 3.7%–4.5% (dry basis). Water used in this study was purified by a Millipore-Q system (Millipore Corp., Saint-Quentin, France). The standard volatile compounds were purchased from Sigma-Aldrich (Steinheim, Germany). Dichloromethane, sodium sulfate and 4-nonanol were obtained from Merck (Darmstad, Germany). Dichloromethane was freshly distilled prior to use.

### 2.2. Extraction of Volatile Compounds

Volatiles of herbs were extracted by the purge and trap system which comprises a flow-meter which controls a nitrogen source and is connected to a splitter system to divide the flow into several channels in order to purge three samples at the same time. Lichrolut EN tubes obtained from Merck were used as an adsorbent which is one of most appropriate sorbents for volatile compounds extraction with respect to the previous research [11]. The herb samples were previously mortared and placed into a 20 mL vial; then, the sample was pre-incubated at optimized purging temperature (60 °C) for 10 min. The process was applied for 90 min with a nitrogen flow of 500 mL/min. After purging, the volatiles held in the cartridge were eluted with dichloromethane. The elute was dried by anhydrous sodium sulphate; the pooled organic extract was concentrated to 5 mL in a Kuderna Danish concentrator fitted with a Snyder column at 40 °C (Supelco, St. Quentin, France) and then to 0.5 mL under a gentle flow of nitrogen. Extracts were then stored at −20 °C in a glass vial equipped with a Teflon-lined cap until analysis. Extractions were carried out in triplicate [12].

### 2.3. GC-FID, GC–MS Analysis of Volatile Compounds

Agilent 6890 chromatograph interfaced with a flame ionization detector (FID) and Agilent 5973-Network-mass selective detector (MSD) (Wilmington, Delaware, DE, USA) constituted the gas chromatography (GC) system. DB-Wax column (30 m length × 0.25 mm i.d. × 0.5 µm thickness, J&W Scientific, Folsom, CA, USA) were used to separate volatile compounds. An amount of 3 µL of extract was injected in pulsed splitless (40 psi; 0.5 min) mode. Injector and FID detectors were set at 270 °C and 280 °C, respectively. The flow rate of carrier gas (helium) was 1.5 mL·min^−1^. The conditions of the oven program of the DB-Wax column was 50 °C to 250 °C at 4 °C/min, 10 min hold. As for the mass-selective detector, the identical oven program was used. The MS (electronic impact ionization) conditions were as follows: ionization energy of 70 eV, mass range m/z of 30–300 a.m.u., scan rate of 2.0 scan·s^−1^, interface temperature of 250 °C, and source temperature of 180 °C. The volatile compounds were analyzed in full scan mode and assigned by comparison of their retention index and their mass spectra on the DB-Wax column with those of a commercial spectra database (Wiley 6, NBS 75k) and the instrument’s internal library made through the aforementioned laboratory researches. After identification, the internal standard method with 4-nonanol was used to determine the mean value of volatile compounds and mean values (µg 100 g^−1^ dry weight; dw) of the triplicate of GC analyses were calculated for each sample. By using *n*-alkane (C_8_–C_32_) series, retention indices of the compounds were calculated [12,13].

## 3. Results and Discussion

**Figure 1:**
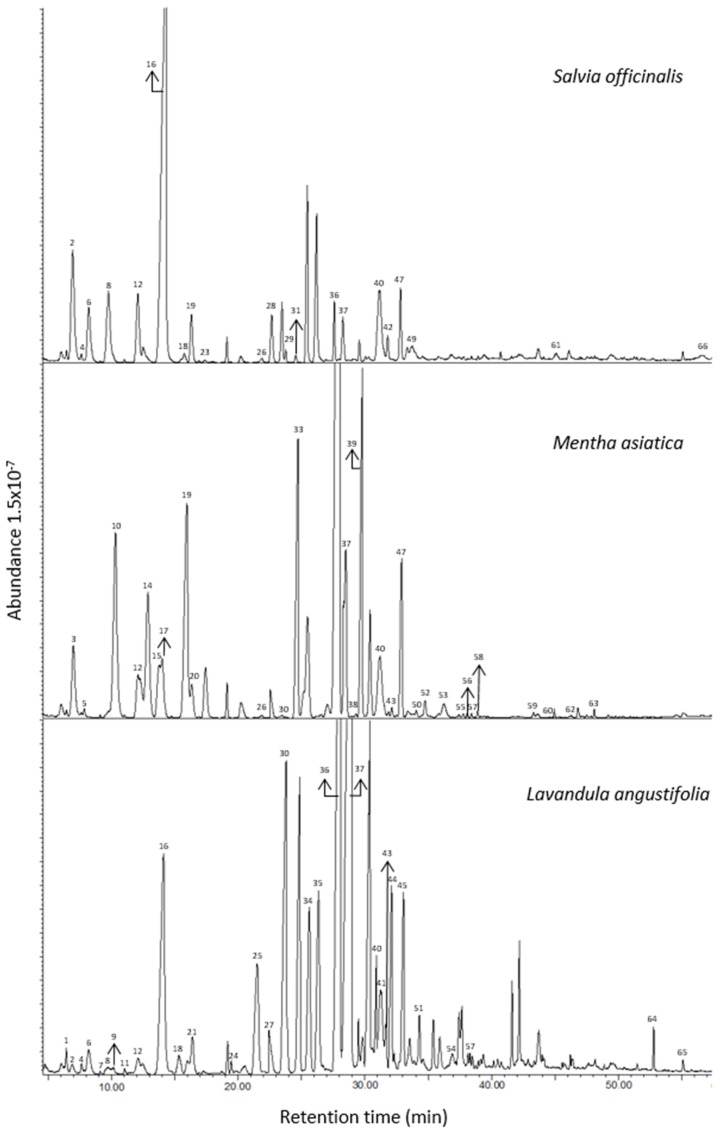
The gas chromatography–mass spectrometry (GC–MS) chromatograms of *Salvia officinalis Lavandula angustifolia* and *Mentha asiatica* (Peak numbers refer to Table 1).

GC–MS investigation of the volatiles extracted from three members of the *Lamiaceae* family by employing the purge and trap extraction method allowed the identification of a total of 66 compounds (Figure 1). As shown in Table 1, a total of 23 volatiles were detected in *Salvia officinalis* extract, while in *Lavandula angustifolia* and *Mentha asiatica* 33 volatiles were extracted. These compounds were classified based on their chemical characteristics: acids, alcohols, aldehydes, esters, hydrocarbons and terpenes. Mean values (μg 100 g^−1^ dw) of the triplicate of GC analyses were calculated. The highest concentration was found in *L. angustifolia* (70,695.1 μg 100 g^−1^ dw) followed by *M. asiatica* (470,653 μg 100 g^−1^ dw) and *S. officinalis* (45691.0 μg 100 g^−1^ dw) showing the difference of genera on the concentration of compounds detected. When the genera were compared, the major variance was detected at the mean values of volatiles in the *L. angustifolia*, which was greater than in *M. asiatica* and *S. officinalis*.

Among the aroma compounds, terpenes were quantitatively and qualitatively the most abundant volatiles detected in the three members of the *Lamiaceae* family. Many plants and parts of them are well known with their pleasant odors, spicy tastes or to show pharmacological activities due to the terpene compounds. These specialties are formed predominantly by terpenes. However, producing purposes and biological functions of these compound have not been completely inspected. Many herbs generate terpenes so as to charm insects for pollination or to protect herbs from being eaten by animals [10]. A total of 43 terpenes were detected and quantified in the herb extracts: 17 detected in *S. officinalis*, 18 in *L. angustifolia* and 25 in *M. asiatica*.

Our results are in accordance with MÉNDEZ-TOVAR, et al. [14] who observed the main aroma compound of wild populations of *Labiatae* species as oxygenated monoterpenes. Within these, terpene *β*-myrcene and caryophyllene compounds were the only terpene compounds identified in all studied herbs. The mean value of the terpene compounds in *L. angustifolia* (47,635 μg 100 g^−1^ dw) was higher than in *S. officinalis* (44,055 μg 100 g^−1^ dw) and *M. asiatica* (37,501 μg 100 g^−1^ dw). The main terpene compounds in *S. officinalis* were 1,8-cineole, *α*-pinene and *β*-pinene. The total concentration of these compounds was 21,341 μg 100 g^−1^ dw, 4233 μg 100 g^−1^ dw and 3044 μg 100 g^−1^ dw, respectively, and accounted for 64% of the total terpene compounds identified in *S. officinalis*.

As previously designated, monoterpenes overwhelmingly affected the overall aroma characteristic of the *S. officinalis* by different researchers. Hayouni et al. [15] investigated the oil characterization of Tunusian *S. officinalis*. They found that major constituents were mainly oxygenated monoterpenes. In addition, the researchers pointed out that the major aroma compound of *S. officinalis* is 1,8-cineole with 33.27% of the total compounds being identified. Likewise, research from different locations previously identified these compounds as the major aroma compounds of *S. officinalis* [16,17,18].

Another studied member of *Lamiaceae* was *M. asiatica*. Sabinene together with *γ*-terpinene and 4-terpineol were detected as the major terpene compounds in this herb. These terpenes were identified in *M. asiatica* and different species of *Mentha* spp. from a different location in previous studies [7,19]. Verma et al. [7] reported that the aroma compounds identified in the studied *Mentha* spp. are oxygenated monoterpenes (74.0%) and sesquiterpene hydrocarbons (18.0%) with lower amounts of monoterpene hydrocarbons (2.6%). Terpene synthases are directly responsible for the production of these volatile terpenes.

On the other hand, some of them are formed via modification of the main skeletons of terpene made by terpene synthases by hydroxylation, dehydrogenation, acylation, and other reactions [20]. The last member of *Lamiaceae* studied was *L. angustifolia*. The main terpene compounds of the sample were linalool, 1,8-cineole and (*Z*)-linalool oxide. Similar to our study, previously published studies highlighted that linalool is the most abundant compound in the *L. angustifolia* [21,22,23]. This compound is an oxygenated monoterpene and one of the main compound of essential oils in various aromatic species. These linalool rich species have been used in traditional medical systems since prehistoric times [1]. Furthermore, previous articles pointed out that this compound acts as a reversible competitive inhibitor of acetylcholinesterase, has been an alternative to conventional insecticides and has dose-dependent marked sedative effects on the central nervous system [24,25,26].

Esters were the second most important class of the aroma compounds in the *Lamiaceae* family. Esters compounds have a very wide range of odor and flavoring effects and there are over 200 of these compounds permitted for use in foods. Moreover, these compounds are widely distributed in essential oils and in some instances represent the major constituent. Generally, ester compounds are responsible for the mature and fruity notes [27]. A total of seven esters were identified and quantified in herbs: two in *S. officinalis*, five in *L. angustifolia* and three in *M. asiatica*. Linalyl acetate was the only compounds which was detected in all samples. This compound is one of the major compounds that characterized the overall aroma of the *L. angustifolia* [22]. Linalyl acetate is a significant compound in the perfume industry and is found in large amounts in various plants [28].

Regarding the other compounds, in trace amounts, acids, alcohols, aldehydes and hydrocarbons were also identified and quantified in the three samples. These compounds account for 0.01%, 0.03% and 0.01% of total aroma compounds, which were identified in *S. officinalis*, *L. angustifolia* and *M. asiatica*, respectively. Most of these volatiles were previously identified in these three members of the *Lamiaceae* family [7,16,17,23,24,29].

## 4. Conclusions

In the present paper, the aim was to determine the aroma compounds of three members of the *Lamiaceae*, *Salvia officinalis*, *Lavandula angustifolia* and *Mentha asiatica*, cultivated in the Turkey. A total of 23, 33, and 33 aroma compounds were identified in *Salvia officinalis Lavandula angustifolia* and *Mentha asiatica*, respectively including, acids, alcohols, aldehydes, esters, hydrocarbons, and terpenes. Terpene compounds were determined as the main chemical group among the identified aroma compounds, followed by esters. A total of 17 terpene compounds were identified in *S. officinalis*, 18 in *L. angustifolia* and 25 in *M. asiatica.* Linalyl acetate was the only and most important ester compound which was detected in all samples.

## Figures and Tables

**Table 1:** Aroma compounds of *Salvia officinalis Lavandula angustifolia* and *Mentha asiatica*.

No	LRI *	Chemical Class	Sum Formula	Compounds	Concentration (μg 100 g^−1^ dw) ^#^			Identification ^§^
					*Salvia officinalis*	*Lavandula angustifolia*	*Mentha asiatica*	
1	1008	Alcohol	C_5_H_10_O	2-Methyl-3-buten-2-ol	nd	309 ± 9.27	nd	LRI, MS, tent
2	1027	Terpene	C_10_H_16_	*α*-Pinene	4233 ± 97.3	240 ± 5.52	nd	LRI, MS, std
3	1038	Terpene	C_10_H_16_	*α*-Thujene	nd	nd	2290 ± 38.9	LRI, MS, tent
4	1075	Ester	C_6_H_12_O_2_	*n*-Butyl acetate	178 ± 6.05	169 ± 5.74	nd	LRI, MS, tent
5	1082	Aldehyde	C_6_H_12_O	Hexanal	nd	nd	304 ± 12.7	LRI, MS, std
6	1087	Terpene	C_10_H_16_	Camphene	2098 ± 52.4	681 ± 17.0	nd	LRI, MS, std
7	1108	Alcohol	C_4_H_10_O_2_	1-Methoxy-2-propanol	nd	38.9 ± 1.32	nd	LRI, MS, tent
8	1124	Terpene	C_10_H_16_	*β*-Pinene	3044 ± 124	208 ± 8.52	nd	LRI, MS, std
9	1130	Hydrocarbon	C_8_H_10_	*m*-Xylene	nd	137 ± 2.87	nd	LRI, MS, std
10	1134	Terpene	C_10_H_16_	Sabinene	nd	nd	7091 ± 219	LRI, MS, std
11	1153	Alcohol	C_5_H_10_O	3-Penten-2-ol	75.3 ± 2.25	91.5 ± 2.74	nd	LRI, MS, tent
12	1167	Terpene	C_10_H_16_	*β*-Myrcene	2573 ± 59.1	519 ± 11.9	1688 ± 38.8	LRI, MS, std
13	1072	Terpene	C_10_H_16_	Camphene	2881 ± 48.9	nd	275 ± 4.67	LRI, MS, std
14	1178	Terpene	C_10_H_16_	*α*-Terpinene	nd	nd	3788 ± 128	LRI, MS, std
15	1190	Terpene	C_10_H_16_	dL Limonene	nd	nd	1537 ± 64.5	LRI, MS, std
16	1199	Terpene	C_10_H_18_O	1,8-Cineole	21341 ± 533	6160 ± 154	nd	LRI, MS, tent
17	1225	Terpene	C_10_H_16_O	*β*-Thujene	nd	nd	1485 ± 50.4	LRI, MS, tent
18	1260	Terpene	C_10_H_16_	*β*-Ocimene	217 ± 8.89	453 ± 18.5	nd	LRI, MS, tent
19	1265	Terpene	C_10_H_16_	*γ*-Terpinene	919 ± 19.2	nd	6588 ± 138	LRI, MS, std
20	1272	Terpene	C_10_H_14_	*o*-Cymene	nd	nd	701 ± 21.7	LRI, MS, std
21	1280	Terpene	C_10_H_14_	*p*-Cymene	nd	683 ± 20.4	nd	LRI, MS, std
22	1285	Aldehyde	C_6_H_10_O	2-Hexanal	nd	27.3 ± 0.62	nd	LRI, MS, std
23	1290	Terpene	C_10_H_16_	*α*-Terpinolene	54.4 ± 0.92	nd	1250 ± 21.2	LRI, MS, std
24	1321	Alcohol	C_6_H_14_O	Hexanol	nd	179 ± 6.08	nd	LRI, MS, std
25	1360	Ester	C_10_H_20_O_3_	1-Octenol acetate	nd	2627 ± 110	nd	LRI, MS, tent
26	1372	Aldehyde	C_9_H_18_O	Nonanal	128 ± 3.2	nd	79.4 ± 1.98	LRI, MS, std
27	1402	Acid	C_2_H_4_O_2_	Acetic acid	nd	665 ± 22.6	406 ± 13.8	LRI, MS, std
28	1415	Terpene	C_10_H_16_O	*β*-Thujone	1008 ± 41.3	nd	nd	LRI, MS, tent
29	1422	Terpene	C_10_H_16_O	*α*-Thujone	113 ± 2.37	nd	nd	LRI, MS, tent
30	1436	Terpene	C_10_H_18_O_2_	(*E*)- linalool oxide	nd	5448 ± 168	60.6 ± 1.87	LRI, MS, tent
31	1445	Alcohol	C_8_H_16_O	1-Octen-3-ol	178 ± 5.34	nd	nd	LRI, MS, tent
32	1456	Terpene	C_10_H_18_O_2_	Epoxylinalool	nd	469 ± 10.7	nd	LRI, MS, std
33	1466	Ester	C_10_H_18_O	Sabinene hydrate	nd	nd	5224 ± 88.8	LRI, MS, tent
34	1495	Terpene	C_10_H_18_O_2_	(*Z*)-Linalool oxide	nd	3809 ± 129	nd	LRI, MS, tent
35	1515	Terpene	C_10_H_16_O	Camphor	nd	2769 ± 116	nd	LRI, MS, std
36	1548	Terpene	C_10_H_18_O	Linalool	755 ± 18.8	19773 ± 494	nd	LRI, MS, std
37	1564	Ester	C_12_H_20_O_2_	Linalyl acetate	700 ± 23.8	13075 ± 384	3443 ± 117	LRI, MS, tent
38	1596	Ester	C_12_H_20_O_2_	*α*-Fenchyl acetate	nd	nd	45.7 ± 1.87	LRI, MS, tent
39	1603	Terpene	C_10_H_18_O	4-Terpineol	nd	nd	5389 ± 113	LRI, MS, std
40	1315	Terpene	C_15_H_24_O	Caryophyllene	2385 ± 73.9	1605 ± 49.7	1949 ± 60.4	LRI, MS, std
41	1625	Acid	C_4_H_8_O_2_	Butyric acid	nd	844 ± 25.32	nd	LRI, MS, std
42	1638	Terpene	C_10_H_18_O	*Δ*-*T*erpineol	428 ± 9.84	nd	nd	LRI, MS, tent
43	1655	Terpene	C_15_H_24_	*β*-Farnesene	nd	550 ± 9.35	78.7 ± 1.33	LRI, MS, tent
44	1663	Ester	C_12_H_20_O_2_	Lavandulyl acetate	nd	4241 ± 144	nd	LRI, MS, std
45	1685	Terpene	C_10_H_18_O	Lavandulol	nd	1892 ± 79.4	nd	LRI, MS, tent
46	1700	Terpene	C_10_H_18_O	(*Z*)-Piperitol	nd	nd	130 ± 3.25	LRI, MS, tent
47	1714	Terpene	C_10_H_18_O	*α*-Terpineol	1093 ± 37.1	nd	2135 ± 72.5	LRI, MS, std
48	1720	Terpene	C_10_H_18_O	Borneol	nd	1731 ± 70.9	nd	LRI, MS, std
49	1723	Terpene	C_15_H_24_	*α*-Humulene	687 ± 14.4	nd	nd	LRI, MS, tent
50	1727	Terpene	C_10_H_14_O	*d*-Carvone	nd	nd	114 ± 3.53	LRI, MS, std
51	1735	Ester	C_12_H_20_O_2_	Neryl acetate	nd	237 ± 7.11	nd	LRI, MS, tent
52	1742	Terpene	C_10_H_18_O	(*E*)-piperitol	nd	nd	273 ± 6.27	LRI, MS, tent
53	1755	Terpene	C_15_H_24_	Bicyclogermacrene	nd	nd	382 ± 6.49	LRI, MS, tent
54	1769	Terpene	C_15_H_24_	*α-F*arnesane	nd	513 ± 17.4	nd	LRI, MS, std
55	1808	Aldehyde	C_10_H_16_O	2-Decadienal	nd	nd	53.4 ± 2.24	LRI, MS, tent
56	1820	Terpene	C_10_H_12_O	Anethole	nd	nd	39.4 ± 0.98	LRI, MS, std
57	1835	Terpene	C_10_H_14_O	*p*-Cymen-8-ol	nd	126 ± 4.28	38.9 ± 1.32	LRI, MS, std
58	1860	Terpene	C_10_H_16_O_3_	Ascaridole	nd	nd	66.4 ± 2.72	LRI, MS, tent
59	1954	Terpene	C_15_H_24_O	Caryophyllene oxide	nd	nd	64.6 ± 1.35	LRI, MS, tent
60	2028	Acid	C_8_H_16_O_2_	Octanoic acid	nd	nd	7.51 ± 0.23	LRI, MS, std
61	2102	Terpene	C_15_H_24_O	Viridiflorol	219 ± 6.57	nd	nd	LRI, MS, tent
62	2162	Terpene	C_10_H_14_O	Thymol	nd	nd	31 ± 0.71	LRI, MS, std
63	2219	Terpene	C_10_H_14_O	Carvacrol	nd	nd	49.8 ± 0.84	LRI, MS, std
64	2450	Hydrocarbon	C_9_H_6_O_2_	Coumarin	nd	229 ± 7.78	nd	LRI, MS, tent
65	2930	Acid	C_16_H_32_O_2_	Palmitic acid	nd	183 ± 7.78	nd	LRI, MS, std
66	3184	Acid	C_18_H_34_O_2_	Oleic acid	374 ± 9.35	nd	nd	LRI, MS, std

* LRI, linear retention index calculated on a DB-WAX capillary column; **^#^** Concentration: Results are the means of three repetitions as µg 100 g^−1^ dw; **^§^** Identification: Methods of identification; LRI (linear retention index), MS tent. (tentatively identified by MS), Std (chemical standard); When only MS or LRI is available for the identification of a compound, it must be considered as an attempt of identification. nd (not detected).
